# Dominance of community-associated methicillin-resistant *Staphylococcus aureus* clones in a maternity hospital

**DOI:** 10.1371/journal.pone.0179563

**Published:** 2017-06-22

**Authors:** Edet E. Udo, Noura Al-Sweih

**Affiliations:** Department of Microbiology, Faculty of Medicine, Kuwait University, Jabriya, Kuwait; Universitatsklinikum Munster, GERMANY

## Abstract

**Background:**

Methicillin- resistant *Staphylococcus aureus* (MRSA) is a major pathogen causing healthcare- and community- acquired infections. The purpose of this study was to characterize MRSA isolated at the Maternity Hospital between 2006 and 2011 for their genetic relatedness.

**Materials and methods:**

The MRSA isolates were investigated using a combination of antibiogram, Staphylococcal chromosome cassette *mec* (SCC*mec*) and spa typing to determine their relatedness to MRSA isolated in other Kuwait hospitals. The isolates were also investigated for the carriage of genes for Pantone valentine Leukocidin (PVL).

**Results:**

A total of 103 MRSA obtained from 64 neonates, 17 adult patients and 12 healthcare workers. The isolates were resistant to Kanamycin (46.6%), gentamicin (40.8%), trimethoprim (32%), ciprofloxacin (22.3%), fusidic acid (16.5%), tetracycline (19.4%), erythromycin (15.5%), clindamycin (15.5%), streptomycin (11.6%) high-level mupirocin (2.9%) and chloramphenicol (0.9%). Twenty (19.4%) of the isolates were multiresistant. Thirty-one (30.0%) isolates were positive for PVL. Molecular typing revealed the presence of 11 clonal complexes and 23 clones with ST5-V-t002, (N = 22), ST22-IV-t223 (N = 18), ST22-IV-t852 (N = 10), ST80-IV-t044 (N = 7), ST5-V-t688 (N = 5), ST772-V-t657 (N = 5) and ST239-III-t860 (N = 4) constituting 66.9% of the isolates. Other clones were isolated sporadically. The number of MRSA isolates increased from two in 2006 to 22 in 2011 with a peak of 43 in 2008.

**Conclusion:**

The study revealed a high prevalence of community-associated MRSA Maternity hospital. The MRSA population consisted of known strains, such as ST239-III-t680, ST22-IV-t223/t852 and ST80-IV-t044, that were reported previously in Kuwait and novel strains such as ST5-V-t002, and several sporadic strains obtained for the first time in the Maternity hospital. This study has provided an initial data which will serve as a platform for future comparative studies on the distribution of MRSA clones in the Maternity hospital in Kuwait.

## Introduction

Methicillin-resistant *Staphylococcus aureus* (MRSA) is a major cause of healthcare- as well as community-acquired infections [1, 2].

Methicillin resistance is determined by the production of an altered low affinity penicillin-binding protein (PBP) called PBP2a (or PBP2’) [3, 4]. PBP2a is encoded by the *mecA* gene, which confers resistance to all beta-lactam antibiotics and is located on a mobile genetic island called staphylococcal cassette chromosome *mec* (SCC*mec*). In addition, to *mecA*, SCC*mec* contains site-specific cassette chromosome recombinases (ccr) that is responsible for the integration of the SCC*mec* into the *S*. *aureus* genome. The combination of SCC*mec*—and recombinase gene complexes defines SCC*mec* types, which differ in size and structural organizations [4,5]. The differences in SCCm*ec* types have been used to type MRSA for epidemiological purposes and to distinguish healthcare-associated MRSA (HA-MRSA) from community-associated MRSA (CA-MRSA). Genetically, CA-MRSA harbor the staphylococcal cassette chromosome *mec* (SCC*mec*) type IV or type V [5], VI or VII [6] whereas HA-MRSA carries SCC*mec* types I, II and III [4, 5].

With the advent of CA-MRSA, infections caused by MRSA have increased in the community settings globally [7]. In addition, the prevalence of MRSA infections and colonization among pregnant women have increased [8, 9, 10]. Similarly, the number of reports linking MRSA with outbreaks in newborn nurseries and neonatal intensive care units are also increasing [11, 12, 13, 14, 15].

The Maternity hospital in Kuwait is a 500-bed tertiary hospital which handles 12000–14000 deliveries, accounting for approximately 30% of all deliveries, in the State of Kuwait [16]. MRSA was not reported in the Maternity Hospital in Kuwait prior to 2005 [17]. This was attributed to its select patient’s population. It was argued that pregnant women admitted to the hospital for child delivery were usually not sick, and spent few days in the hospital after delivery thereby eliminating the risk factor for the acquisition and transmission of healthcare—associated MRSA. However, the situation has since changed and the number of MRSA isolated at the Maternity hospital has been increasing gradually following the isolation of a single MRSA strain in 2005. Molecular typing of 22 MRSA isolates recovered from babies in the Special babies care unit of the Maternity hospital between 1 October and 31 December 2011, revealed the presence of CA-MRSA clones that were different from those usually isolated at other hospitals in Kuwait [16]. This finding revealed a lack of appreciation of the prevailing MRSA clones and their resistance profiles in other facilities of the hospital and demanded further investigations of MRSA isolated previously in the hospital. Consequently, to fully appreciate the epidemiology of MRSA in the Maternity hospital, MRSA that were isolated between 2006 and 2011 were characterized using a combination of molecular typing methods. Knowledge of the clonal types and their resistance patterns would provide useful background data which will be useful in assessing future changes in the MRSA population as well as assessing effectiveness of infection prevention and control measures designed to prevent MRSA becoming endemic in the hospital.

## Materials and methods

### MRSA isolates

The MRSA isolates were obtained as part of routine diagnostic microbiology testing and later submitted to the MRSA Reference Laboratory for molecular typing. In total, 103 MRSA isolates recovered between 2006 and 2011 from 64 neonates (72 isolates), 17 adult patients (18 isolates) and 12 healthcare workers (13 isolates) were investigated. The MRSA isolates were from 37 skin and soft tissues samples, 13 umbilical cord stumps, 18 nasal swabs, 11 eye swabs, 7 blood samples, 4 Vaginal swabs, and 13 miscellaneous sources. The MRSA isolates were identified using standard bacteriological methods including growth on Mannitol Salt Agar, Gram stain, tube coagulase and DNase testing and preserved in 40% glycerol (v/v) in nutrient broth at -80°C.

### Antibiotic susceptibility testing

Susceptibility to benzyl penicillin, cefoxitin, kanamycin, gentamicin, erythromycin, clindamycin, chloramphenicol, tetracycline, minocycline, trimethoprim, fusidic acid, rifampicin, ciprofloxacin, mupirocin and linezolid was determined by the disk diffusion method [18]. Susceptibility to fusidic acid was interpreted following the British Society for antimicrobial chemotherapy guidelines [19]. Minimum inhibitory concentration (MIC) was determined for cefoxitin, mupirocin, vancomycin, and teicoplanin with Etest strips (BioMerieux, Marcey l’ Etoile, France) and interpreted following guidelines by the Clinical and Laboratory Standards Institute [18]. MIC for vancomycin was performed by the Etest macro method per the manufacturer’s protocol. *S*. *aureus* strain ATCC25923 was used as quality control strain for susceptibility testing. Methicillin resistance was confirmed by detecting PBP 2a in culture supernatants using a rapid latex agglutination kit (Denka-Seiken, Japan) following the manufacturer’s instruction. Multiple resistance was defined as resistance to three or more classes of antibacterial agents.

### Detection of genes coding for Panton-Valentine leukocidin, (PVL)

The LukS-PV- lukF-PV gene which codes for PVL was amplified as described previously [20].

### SCCmec typing and subtyping

SCC*mec* typing was performed by PCR assays as described previously [21, 22].

### Spa typing

Spa typing was performed as described by Harmsen *et al*. [23] for all MRSA isolates. DNA sequencing was performed using a 3130x1 genetic analyzer (Applied Bio systems, Forster City, CA. USA) in accordance with the manufacturer protocol. Isolates were assigned to spa types using the spa typing website (http://www.spaserver.ridom.de) [24].

### Multilocus sequence typing (MLST)

MLST was performed on all 37 isolates as described by Enright *et al*., [25]. Isolates were assigned a sequence type (ST) according to the MLST website (http://www.mlst.net) [26]. Clonal complexes were defined as group of sequence types (STs) that share at least five of seven identical alleles with at least one of the STs in the group [27].

## Results

### Antibiotic susceptibility

All MRSA isolates were susceptible to vancomycin, (MIC ≤ 2.0 mg/L), teicoplanin (MIC ≤ 2.0 mg/L), linezolid and rifampicin but were resistant to penicillin G (N = 93; 90.3%), gentamicin (N = 42; 40.8%), kanamycin (N = 48; 46.6%), trimethoprim (N = 33; 32.0%), ciprofloxacin (N = 23; 22.3%), fusidic acid (N = 17;16.5%), tetracycline (N = 20;19.4%), erythromycin (N = 16;15.5%), clindamycin (N = 16;15.5%), streptomycin (N = 12;11.6%), high-level mupirocin (N = 3; 2.9%) and chloramphenicol (N = 1; 0.9%).

Thirty-one (30.0%) isolates obtained from skin and soft tissue infections (13 isolates), nasal swabs (2 isolates), groin (2 isolates), HVS (3 isolates), and one each from blood, eye swab, catheter tip and unspecified source were positive for genes encoding PVL.

### Molecular typing of MRSA isolates

Molecular typing yielded three SCC*mec* types (III, IV and V), 28 spa types, 19 sequence types (ST), 23 clones and 11 clonal complexes (CC) (Table 1). Fifty-seven (55.3%) of the isolates carried SCC*mec* IV genetic element, followed by SCC*mec* V (40 isolates, 38.8%) and SCC*mec* III (6 isolates, 5.8%). SCC*mec* types I and II were not detected.

**Table 1:** Characteristics of MRSA genotypes: 2006–2011.

CC	MRSA Clones	#	PVL	Antibiotic resistance Profiles (# of resistant strains)
**CC1**	ST772-V-t657	1	-	-
	ST772-V-t657	4	+	Gm (4), Km (4), Tp (4), Cip (1)
**CC5**	ST5-V-t002	22	-	Gm (22), Km (22), Tp (2), Fa (2)
	ST5-V-688	5	-	Em (2), Clin (2), Cm (1), Tet (5)
	ST5-IVa-t214	1	-	Cip
	ST5-V-t985	1	-	Em, Clin, LR-MUP
	ST5-IV-688	1	-	-
	ST5-IV-t002	1	+	Em, Clin, Cip
	ST194-IV-t002	1	-	-
	ST627-IV-t105	1	+	Tp
	ST1317-V-t311	1	-	Em, Clin, Tp, Fa, Cip
	ST1462-IV-t690	2	+	Tet
	ST1462-IV-t16184	1	+	Em, Clin
**CC6**	ST932-IV-t304	7	-	-
**CC8**	ST239-III-t860	4	-	Gm (4), Km (4), Sm (4), Em (4), Clin (4), Tet (4), Fa (4), Cip (4), LR-Mup (1), HLR-MUP (3)
	ST239-III-t16202	1		Gm, Km, Em, Clin, Tp, Cip
	ST241-III-t037	1	-	Gm, Km, Sm, Em, Clin, Tp, Cip
**CC9**	ST9-V-t267	1	-	Gm, Km, Fa
**CC22**	ST22-IV-t005	1	+	Km, Em, Clin, Tet, Fa, Cip, LR-MUP
	ST22-IVa-t223	18	-	Te (3), Tp (12), Km (2), Fa (2), Gm (1), Em (1), Clin (1), Cip (1), Sm (1).
	ST22-IV-t15316	1	-	Tp, Cip
	ST22-IV-t852	9	+	Gm (6), Km (6), Tp (5), Cip (10)
	ST60-IV-t16202	1	-	-
**CC30**	ST30-IVa-t318	1	+	-
	ST77-IV-t019	1	+	-
	ST1055-IVa-t2051	1	+	Tp
**CC80**	ST80-IV-t044	7	+	Km (4), Sm (6), Tet (4), Fa (4), Tp (3), Em (1), Clin (1)
	ST80-IV-t376	1	-	Em, Clin
**CC88**	ST2148-IV-t6728	1	-	Tet, Tp
**CC97**	ST97-V-t1234	2	-	Tet (1)
	ST97-V-t359	2	-	Gm (2), Km (2), Fa (2)
**CC121**	ST121-V-t314	1	+	Tet

The major spa types were t002 (N = 24), t223 (N = 18), t852 (N = 10), t044 (N = 7), t304 (N = 7), t688 (N = 6), t657 (N = 5), t860 (N = 4), t1234 (N = 2), t16202 (N = 2), t359 (N = 2) and t690 (N = 2). Spa types, t005, t019, t037, t105, t15316, t16184, t2051, t267, t311, t314, t318, t985, t376, t6728 occurred in single isolates. ST5 (N = 31) and ST22 (N = 29) were the dominant sequence types with both accounting for 58.2% of the isolates. Other common STs were ST80 (N = 8; 7.7%), ST932 (N = 7; 6.8%), ST772 (N = 5; 4.8%), ST239 (N = 5; 4.8%) and ST97 (N = 4; 3.8%).

The dominant clonal complexes were CC5 (N = 37), CC22 (N = 30), CC80 (N = 8), CC6 (N = 7), CC8 (N = 6) and CC1 (N = 5).

CC1 consisted of five ST772-V-t657 isolates. Four of these isolates were positive for PVL and were resistant to gentamicin, kanamycin, and trimethoprim. One isolate was negative for PVL and was susceptible to the non-beta-lactam antibiotics tested. The isolates were obtained from three babies (3 isolates) and adult patients (2 isolates) (Table 2).

**Table 2:** MRSA isolates obtained from adult patients.

	Strain No	Year	Sample	MRSA Genotype
**1**	6186	2007	Wound	ST932-IVa-t304
**2**	6462	2007	**S/N**	ST22-IVa-t223
**3**	6602	2007	Nasal	ST241-III-t037
**4**	6708	2007	Anal swab	ST932-IV-t304
**5**	7016	2008	Wound	ST772-V-t657
**6**	7137	2008	Sputum	ST5-IV-t688
**7**	7149	2008	HVS	ST80-IV-t044
**8**	7231	2008	Wound	ST22-IV-t223
**9**	7800	2008	Wound	ST80-IV-t044
**9**	7858	2008	HVS	ST80-IV-t044
**10**	7890	2008	HVS	ST772-V-t657
**11**	7991	2008	Abscess	ST1462-IV-t16184
**12**	7948	2008	Nasal	ST5-V-t002*
**13**	7969	2008	Wound	ST1055-IV-t2051
**14**	7434	2008	NS	ST97-V-t1234
**15**	7470	2008	Wound	ST5-IV-t002
**16**	7509	2008	Wound	ST22-IV-t15316
**17**	10561	2011	NS	ST932-IV-t304*

*; identical to isolates from their babies.

NS; not specified; HVS, high vaginal swab.

CC5 comprised diverse clones with ST5-V-t002 as the dominant clone, followed by ST5-V-t688. The ST5-V-t002 isolates were negative for PVL, but were resistant to gentamicin and kanamycin and variable resistance to other antibiotics (Table 1). ST5-V-t002 was widely distributed and was isolated from babies, adult patients (Table 2) and healthcare workers (Table 3). All five ST5-V-t688 isolates were resistant to tetracycline but expressed variable resistance to erythromycin, clindamycin and chloramphenicol. ST5-V-t688 isolates were recovered from babies, adult patients (Table 2) and healthcare workers (Table 3).

**Table 3:** MRSA isolates obtained from healthcare workers.

Occupation	Source	MRSA clone
Receptionist 1	Wound	ST80-IV-t044
Lab. Technologist 1	Nasal	ST22-IVa-t223
Respiratory Technician	Wound	ST22-IVa-t223
Lab. Technologist 2	HVS	ST80-IV-t044
Receptionist 2	Wound	ST1462-IV-t690
Receptionist 2	HVS	ST1462-IV-t690
Receptionist 3	Wound	ST22-IV-t223
Receptionist 4	Wound	ST932-IV-t304
Nursing Staff1	Nasal	ST5-V-t002
Nursing Staff3	Nasal	ST5-V-t688
Nursing Staff7	Nasal	ST5-V-t002
Nursing Staff 19	Nasal	ST22-IVa-t223
Nursing Staff 26	Nasal	ST5-V-t002

CC6 consisted of seven ST932-IV-t304 isolates that were negative for PVL and uniformly susceptible to the non-beta-lactam antibiotics tested. The isolates were obtained from babies, one healthcare worker and three adult patients including a mother and her baby (Table 2).

CC8 consisted of six isolates that belonged to ST239-III-t860 (4 isolates), ST239-III-t16202 (1 isolate) and ST241-III-t037 (1 isolate). The CC8 isolates were negative for PVL and expressed multi antibiotic resistance. The t860 isolates were resistant to high-level mupirocin. ST239-III isolates were recovered only from babies while the ST241-III-t037 isolates was obtained from an adult patient (Table 2).

CC22 consisted of ST22- MRSA isolates carrying SCC*mec* IV genetic elements, and belonged to four different spa types, t005, t223, t15316 and t852 with t223, detected in 18 isolates, as the dominant spa type. The ST22 isolates differed in the carriage of genes for PVL and antibiotic resistance (Table 1). The ST22-IV-t005 and most of the ST22-IV-t852 isolates expressed multi antibiotic resistance whereas the ST22-IVa-t223 isolates were susceptible to most of the non-beta-lactam antibiotics. Whereas the t005 and t852 isolates were positive for PVL and negative for toxic shock syndrome toxin gene, the t223 isolates were negative for PVL but positive for toxic shock syndrome toxin gene. The ST22-IVa-t223 isolates were obtained from colonization as well as infection sites of babies, adult patients (Table 2) and healthcare workers (Table 3). The t852 isolates were isolated from babies and a baby cot.

CC30 was represented by three single clones, ST30-IVa-t318, ST77-IV-t019 and ST1055-IV-t2051 that were positive for PVL and susceptible to most non-beta-lactam antibiotics.

CC80 consisted of ST80-IV-t044 (7 isolates) that were positive for PVL and variably resistant to kanamycin, tetracycline, fusidic acid, trimethoprim, erythromycin and clindamycin, and ST80-IV-t376 (1isolate) that was negative for PVL and resistant to erythromycin and clindamycin only. ST80-IV-t044 isolates were obtained from babies, adult patients (Table 2) and healthcare workers (Table 3). CC9, CC88 and CC121 consisted of single isolates while CC97 consisted of four isolates comprising two ST97-V-t1234 and ST97-V-t359 (Table 1).

### Distribution of MRSA isolates by year

**Fig 1:**
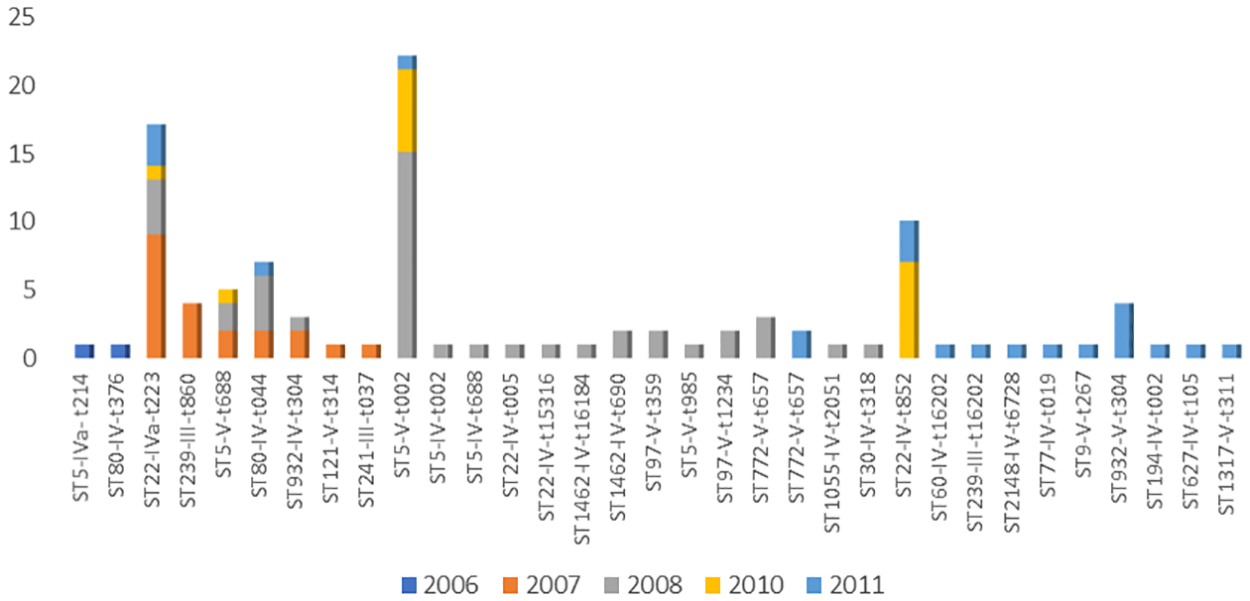
Distribution of MRSA clones by year: 2006–2011.

The isolates were analyzed by year of isolation to ascertain if any of the clones persisted in any units of the hospital. Fig 1 presents the MRSA isolates by year of isolation. Number of isolates increased from two in 2006 to a peak of 43 in 2008 with most clones occurring sporadically. The dominant clones, ST5-V-t002 and ST22-IVa-t223, were isolated in multiple years in clusters. Fifteen of the 22 ST5-V-t002 isolates were obtained in 2008. Eleven of these isolates were obtained from babies and a mother in the same ward depicting local transmission. The remaining seven isolates were obtained in 2010 (6 isolates) and 2011 (1 isolate) from four different wards. The six ST5-V-t002 isolates obtained in 2010 were all from babies in one ward. ST5-V-t688 isolates were obtained in 2007 (2 isolates), 2008 (2 isolates), and 2010 (1 isolate).

The ST22-IV isolates, obtained in 2007, 2008, 2010 and 2011, carried different spa types. Most (9/18) of the ST22-IVa-t223 isolates were obtained in 2007 while four and three of the isolates were obtained in 2008 and 2011 respectively. Six of the 10 ST22-IV-t852 isolates were recovered in 2010 from five babies and a baby cot in the same ward. The other three isolates were obtained in 2011. The ST80-IV-t044 isolates were all obtained in 2007 (2 isolates), 2008 (4 isolates) and 2011 (1 isolate).

ST932-IV-t304 were isolated in 2007 (2 isolates), 2008 (1 isolate) and 2011 (4 isolates). The ST772-V-t657 were isolated in 2008 (3 isolates) and 2011 (2 isolates).

## Discussion

As the epidemiology of MRSA continue to change, the emergence of novel clones with novel virulence factors necessitates regular surveillance for better infection control and management of infection. Molecular typing revealed that the MRSA population in the Maternity hospital belonged to diverse genetic backgrounds dominated by internationally recognized clonal complexes CC5, CC22, CC80, CC6, CC8, CC1 and CC97. The major clones were ST5-V-t002 (21.3%), ST22-IVa-t223 (16.5%), ST22-IV-t852 (9.7%), ST932-IV-t304 (6.7%), ST80-IV- t044/t376 (7.7%), ST772-V-657 (4.8%) and ST239-III-t860 (3.8%). Apart from ST239-III/ST241-III and ST22-IV, which are healthcare- associated MRSA clones, the majority (66.0%) of the isolates had community-associated MRSA (CA-MRSA) genotypes. The high prevalence of CA-MRSA observed in this study mirrors recent observations in other centers where CA-MRSA are the dominant MRSA isolates obtained from neonatal units [12, 28, 29, 30, 31, 32].

This study also revealed that the MRSA population consisted of strains that have been reported previously in other hospitals in Kuwait, and those reported for the first time at the Maternity hospital. Isolates reported previously at other hospitals include ST239-III-t860, ST241-III-t037, ST5-IV-t688, ST22-IV-t223/t852, ST80-IV-t044/t376, ST772-V-657, ST121-V-t314, ST5-IV-t002 and ST97-V-t1234 [33, 34]. Unexpectedly, ST239-III-MRSA, which is the dominant MRSA clone encountered in other Kuwait hospitals [33], was present in small numbers in this study. Secondly, the ST239-III-t860 isolates, obtained only in 2007, did not persist in the Maternity hospital in contrast to their persistence in other hospitals in Kuwait [33, 35]. Most of the ST22-IVa-t223 isolates were obtained from samples of skin and soft tissue infections as well colonization sites as was the case in other hospitals in Kuwait [36] supporting their ability to be involved in different types of infections. In contrast, the UK-EMRSA-15/Barnim epidemic MRSA clone is mostly associated with bloodstream infections [37]. The detection of the *tst-*positive ST22-IVa-t223 isolates among different patients’ populations in this hospital suggests that it is becoming an important epidemic healthcare- associated pathogen in Kuwait hospitals. In addition to its growing prominence in Kuwait hospitals, *tst-*positive ST22-IVa-t223 isolates have also been reported widely in the Gaza strip [38], Jordan [39], Saudi Arabia and Egypt [40] and is becoming the leading variant of the UK Epidemic MRSA-15 clone in the Middle East affecting adults and babies. Besides the *tst*-positive ST22-IVa-t223 isolates, PVL-positive -ST22-IV-t852 MRSA also contributed to the MRSA burden among the neonates. In addition, a t16202 variant of ST22-IV-MRSA was also detected for the first time in this study adding to the ongoing diversification of CC22 strains observed in recent years [36, 41, 42].

The isolation of ST80-IV-MRSA in eight (7.7%) of the isolates in this study is comparable to the prevalence of 7.5% prevalence that was reported recently in other hospitals in Kuwait [33]. However, with only two of the eight isolates obtained from neonates, the results suggest that ST80-IV-MRSA had limited role in infections among the neonates. In contrast, ST80-IV-t044 clone was recently reported as the leading cause of infections among neonates in a hospital in Algeria [29].

Two of the four ST97-V-MRSA isolate, ST97-V-t359 isolates, were resistant to gentamicin, kanamycin and fusidic acid like the resistance profiles of ST97-V-MRSA isolates that caused an outbreak in a neonatal intensive care unit in another Kuwait hospital in 2007 [12]. The other two ST97-V-t1234 isolates were resistant to tetracycline and resembled isolates reported previously in another Kuwait hospital [33]. ST97-V-MRSA isolates have also been reported sporadically from Jordan [39], Saudi Arabia [43], Lebanon [44] and countries outside the Middle East including Australia [45], USA [46], UK [47], and Brazil [48] underlying their growing importance as a human pathogen in addition to their roles as veterinary pathogens [49, 50].

Novel MRSA clones isolated at the Maternity hospital included the dominant clone, ST5-V-t002, recovered from healthcare workers, adult patients and neonates, and sporadic clones such as ST77-IV-t019, and ST9-V-t267. Although newly isolated in Kuwait, ST5-V-t002 isolates have been reported to cause infections in other countries [42, 51].

Prior to the report of the ST9-V-t267 isolate in this study, CC9 isolates were recovered from animals or farm workers [42]. The present isolate was obtained from a blood sample of a baby admitted to the Special Care unit. However, history of parental contact with animals could not be established.

The isolation of the common MRSA clones, ST5-V-t002, ST22-IVa-t223 and ST80-IV-t044 from neonates, healthcare workers and adult patients in the hospital is suggestive of nosocomial transmission of these clones. The presence of colonized healthcare workers, neonates and their mothers, and adult patients in other wards of the hospital have previously been documented to provide opportunities for nosocomial transmissions of MRSA in a neonatal ward [52]. Furthermore, the isolation of ST5-V-t002 from a mother, her baby and nine other babies in the same unit in 2008 is suggestive of an outbreak with the mother as the likely source of the organism [53]. Likewise, the detection of ST22-IV-t852 from a baby’s cot and five different babies in the same unit suggests that the environment also contributed to the nosocomial transmission [54]. Therefore, multiple sources including colonized mothers, adult patients, healthcare workers and the environment contributed to the MRSA population in the Maternity hospital.

The study further sought to establish whether any of the clones persisted in the hospital overtime. The results indicated that although ST5-V-t002, ST22-IVa-t223 and ST80-IV-t044 were isolated in more than one year, the numbers isolated per year were small which suggested multiple acquisition rather than persistence. The other isolates occurred sporadically supporting their transient acquisition.

Although the isolates were susceptible to vancomycin, teicoplanin, linezolid and rifampicin, their susceptibility patterns followed predictable patterns with HA-MRSA isolates, ST239/ST241 –III-MRSA, ST22-IV-t005 and ST22-IV-t852 MRSA expressing multi resistance whereas the CA-MRSA isolates belonging to CC6 (ST6-IV-t6269, ST932-IV-t304 and ST932-IV-t657) were susceptible to all non-beta-lactam antibiotics and the other CA-MRSA clones were susceptible to most of the non- beta-lactam antibiotics.

In conclusion, the MRSA isolates belonged to diverse genetic backgrounds with ST5-V-t002 and ST22-IVa-t223 as the dominant clones. Multiple sources including healthcare workers, colonized or infected patients, colonized mothers and the environment contributed to the MRSA population which consisted of MRSA clones that have been isolated widely in other Kuwait hospitals and sporadic novel clones. This study has provided an initial data which will serve as a platform for future comparative studies on the distribution of MRSA clones in the Maternity hospital in Kuwait.

## References

[1] Chambers HF (2001) The changing epidemiology of Staphylococcus aureus? Emerg Infect Dis 7: 178–182. 11294701 10.3201/eid0702.010204 PMC2631711

[2] Maltezou H, Giamarellou H (2005) Community-acquired methicillin-resistant *Staphylococcus aureus* infections. Int. J. Antimicrob Agents 27: 87–96. 10.1016/j.ijantimicag.2005.11.004 16423509

[3] Ito T, Katayama Y, Asada K, Mori N, Tsutsumimoto K, Tiensasitoro C, et al (2001) Structural comparison of three types of cassette chromosome *mec* integrated in the chromosome in methicillin-resistant *Staphylococcus aureus*. Antimicrob Agents Chemother 45: 1323–1336. doi: 10.1128/AAC.45.5.1323-1336.2001 11302791 10.1128/AAC.45.5.1323-1336.2001 PMC90469

[4] Ma XX, Ito T, Tiensasitorn C, Jamklang M, Chongtrakool P, Boyle-Vavra, et al (2002) Novel type of staphylococcal cassette chromosome mec identified in community-acquired methicillin-resistant *Staphylococcus aureus* strains. Antimicrob Agents Chemother 46: 1147–1152. doi: 10.1128/AAC.46.4.1147-1152.2002 11897611 10.1128/AAC.46.4.1147-1152.2002 PMC127097

[5] Ito T, Ma XX, Takeuchi F, Okuma K, Yuxawa H, Hiramatsu K (2004) Novel Type V Staphylococcal cassette chromosome *mec* driven by a novel cassette chromosome recombinase, ccrC. Antimicrb Agents Chemother 48: 2637–2651. 10.1128/AAC.48.7.2637-2651.2004 PMC434217 15215121

[6] Higuchi W, Takano T, Teng LI, Yamamoto T (2008) Structure and specific detection of staphylococcal cassette chromosome *mec* type VII. Biochem Biophys Res Commun 377: 752–756. doi: 10.1016/j.bbrc.2008.10.009 18926798 10.1016/j.bbrc.2008.10.009

[7] David M Z, Daum RS (2010) Community-associated methicillin-resistant *Staphylococcus aureus*: epidemiology and clinical consequences of an emerging epidemic. Clin. Microbiol. Rev 23:616–687. doi: 10.1128/CMR.00081-09 20610826 10.1128/CMR.00081-09 PMC2901661

[8] Bratu S, Eramo A, Kopec R, Coughlin E, Ghitan M, Yost R, et al (2005) Community-associated methicillin-resistant *Staphylococcus aureus* in hospital nursery and maternity units. Emerg Infect Dis 11: 808–813. doi: 10.3201/eid1106.040885 15963273 10.3201/eid1106.040885 PMC3367583

[9] Carey AJ, Duchon J, Della-Latta P, Saiman L (2010) The epidemiology of methicillin-susceptible and methicillin-resistant *Staphylococcus aureus* in a neonatal intensive care unit. J Perinatol 30: 135–139. doi: 10.1038/jp.2009.119 19710681 10.1038/jp.2009.119

[10] Creech CB, Litzner B, Talbot TR, Schaffner W (2010) Frequency of detection of methicillin-resistant *Staphylococcus aureus* from rectovaginal swabs in pregnant women. Am J Infect Control 38:72–74. doi: 10.1016/j.ajic.2009.06.015 19836855 10.1016/j.ajic.2009.06.015

[11] Kamath S, Mallaya S, Shenoy S (2010) Nosocomial infections in neonatal intensive care units: profile, risk factor assessment and antibiogram. Indian J Pediatr 77: 37–39. doi: 10.1007/s12098-010-0005-5 20135266 10.1007/s12098-010-0005-5

[12] Udo EE, Aly NY, Sarkhoo E, Al-Sawan R, Al-Asar AS (2011) Detection and characterization of an ST97-SCCmec-V community-associated methicillin-resistant *Staphylococcus aureus* clone in a neonatal intensive care unit and special care baby unit. J Med Microbiol 60: 600–604. doi: 10.1099/jmm.0.028381-0 21292856 10.1099/jmm.0.028381-0

[13] McAdams RM., Ellis MW, Trevino S, Rajnik M (2008) Spread of methicillin-resistant *Staphylococcus aureus* USA300 in a neonatal intensive care unit. Pediatr Int. 6: 810–815. 10.1111/j.1442-200X.2008.02646.x 19067897

[14] Garcia CP, Rosa JF, Cursino MA, Lobo RD, Mollaco CH, Gobara S, et al (2014) Non-multidrug-resistant- methicillin-resistant *Staphylococcus aureus* in a neonatal unit. Peditr Infect Dis 33: e252–9. 10.1097/INF.0000000000000435 24892848

[15] Geraci DM, Giuffre M, Bonura C, Matranga D, Aleo O, Saporito L, et al (2014) Methicillin-resistant *Staphylococcus aureus* colonization: a three-year prospective study in a neonatal intensive care unit in Italy. PloS One 9: e87760 doi: 10.1371/journal.pone.0087760 24505312 10.1371/journal.pone.0087760 PMC3914835

[16] Udo EE, Al-Sweih N (2013) Emergence of methicillin-resistant *Staphylococcus aureus* in the Maternity hospital, Kuwait. Med Princ Pract 22: 535–539. doi: 10.1159/000350526 23635861 10.1159/000350526 PMC5586800

[17] Udo EE, Al-Sweih N, Dhar R, Dimitrov T, Mokaddas EM, Johny M, et al (2008) Surveillance of antibacterial resistance in *Staphylococcus aureus* isolated in Kuwait hospitals. Med Princ Pract 17: 71–75. doi: 10.1159/000109594 18059105 10.1159/000109594

[18] Clinical and Laboratory Standard Institute (2012) Performance standards for antimicrobial susceptibility testing; Twenty-Second Informational Supplement. CLSI document M100-S22, Wayne, PA.

[19] BSAC: BSAC methods for susceptibility testing. Version 9.1. March 2010.

[20] Lina G, Piemont Y, Godail-Gamot F, Bes M, Peter M-O, Gauduchon V, et al (1999) Involvement of Panton-Valentine leukocidin producing *Staphylococcus aureus* in primary skin infections and pneumonia. Clin Infect Dis 29: 1128–1132. doi: 10.1086/313461 10524952 10.1086/313461

[21] Oliveira DC, de Lencastre H (2002) Multiplex PCR strategy for rapid identification of structural types and variants of the *mec* element in methicillin-resistant *Staphylococcus aureus*. Antimicrob Agents Chemother 46: 2155–2161. doi: 10.1128/AAC.46.7.2155-2161.2002 12069968 10.1128/AAC.46.7.2155-2161.2002 PMC127318

[22] Zhang K, McClure J-A, Elsayed S, Louie T, Conly JM (2005) Novel Multiplex PCR Assay for Characterization and Concomitant Subtyping of Staphylococcal Cassette Chromosome *mec* Types I to V in Methicillin-Resistant *Staphylococcus aureus*. J Clin Microbiol 43: 5026–5033. doi: 10.1128/JCM.43.10.5026-5033.2005 16207957 10.1128/JCM.43.10.5026-5033.2005 PMC1248471

[23] Harmsen D, Claus H, Witte W, Rothanger J, Claus H, Turnwald D, et al (2003) Typing of methicillin-resistant *Staphylococcus aureus* in a University Hospital setting by using novel software for spa repeat determination and database management. J Clin Microbiol 41: 5442–5448. doi: 10.1128/JCM.41.12.5442-5448.2003 14662923 10.1128/JCM.41.12.5442-5448.2003 PMC309029

[24] http://www.spaserver.ridom.de.

[25] Enright MC, Day NPJ, Davies CE, Peacock SJ, Spratt BG (2000) Multilocus sequence typing for characterization of methicillin-resistant and methicillin-susceptible clones of *Staphylococcus aureus*. J Clin Microbiol 38: 1008–1015. 10698988 10.1128/jcm.38.3.1008-1015.2000 PMC86325

[26] http://www.mlst.net.

[27] Enright MC, Robinson DA, Randle G, Feil EJ, Grundman H, Spratt BG (2002) The evolutionary history of methicillin-resistant *Staphylococcus aureus* (MRSA). Proc Natl Acad Sci USA 99: 7687–7692. doi: 10.1073/pnas.122108599 12032344 10.1073/pnas.122108599 PMC124322

[28] Machuca MA, Sosa LM, Gonzalez CL (2013) Molecular typing and virulence characteristics of methicillin-resistant *Staphylococcus aureus* isolates from pediatric patients in Bucaramanga, Columbia. PLoS One 8: e73434 doi: 10.1371/journal.pone.0073434 24058415 10.1371/journal.pone.0073434 PMC3751954

[29] Djoudi F, Bonura C, Benallaoua S, Touati A, Touati D, Aleo A, et al (2013) Panton-Valentine leukocidin positive sequence type 80 methicillin-resistant *Staphylococcus aureus* carrying a staphylococcal cassette chromosome mec type IVc is dominant in neonates and children in an Algiers hospital. New Microbiol 36: 49–55. 23435815

[30] Schaumburg F, Alabi AS, Mombo-Ngoma G, Kaba H, Zoleko RM, Diop DA, et al (2014) Transmission of *Staphylococcus aureus b*etween mothers and infants in an African setting. Clin Microbiol Infect 20: 390–396. 10.1111/1469-0691.12417 24118578

[31] Alsubaie S, Bahkali K, Somily AM, Alzamil F, Alrabiaah A, Alaska A, et al (2012) Nosocomial transmission of community-acquired methicillin-resistant *Staphylococcus aureus* in a well-infant nursery of a teaching hospital. Pediatr Int. 54: 786–792. doi: 10.1111/j.1442-200X.2012.03673.x 22640461 10.1111/j.1442-200X.2012.03673.x

[32] Ali H, Nash JQ, Kearns AM, Pichon B, Vasu V, Nixon Z, et al (2012) Outbreak of a South West Pacific clone Panton-Valentine leucocidin-positive meticillin-resistant *Staphylococcus aureus* infection in a UK neonatal intensive care unit. J Hosp Infect 80: 293–298. doi: 10.1016/j.jhin.2011.12.019 22361299 10.1016/j.jhin.2011.12.019

[33] Boswihi SS, Udo EE, Al-Sweih N (2016) Shifts in the clonal distribution of methicillin-resistant Staphylococcus aureus in Kuwait hospitals: 1992–2010. PloS ONE 11: e0162744 doi: 10.1371/journal.pone.0162744 27631623 10.1371/journal.pone.0162744 PMC5025013

[34] Udo EE, Al-Sweih N, Noronha B (2006) Characterization of Non multiresistant methicillin—resistant *Staphylococcus aureus* (including EMRSA-15) in Kuwait Hospitals. Clin Microbiol Infect 12: 262–269. doi: 10.1111/j.1469-0691.2005.01350.x 16451414 10.1111/j.1469-0691.2005.01350.x

[35] Al-Haddad AM, Udo EE, Mokadas EM, Sanyal SC, Grubb WB (2001) Persistence of a clone of methicillin-resistant *Staphylococcus aureus* in a burns unit. J Med Microbiol 50: 558–564. doi: 10.1099/0022-1317-50-6-558 11393293 10.1099/0022-1317-50-6-558

[36] Udo EE, Boswihi SS, Al-Sweih N (2016) High prevalence of toxic shock syndrome toxin- producing methicillin-resistant *Staphylococcus aureus* 15 (EMRSA-15) strains in Kuwait hospitals. New Microbe and New Infect 12: 24–30. 10.1016/j.nmni.2016.03.008 PMC4873690 27222714

[37] Johnson AP, Aucken HM, Cavendish S, Ganner M, Wale MC, Warner M, et al (2001) Dominance of EMRSA-15 and -16 among MRSA causing nosocomial bacteremia in the UK. Analysis of isolates from the European Antimicrobial Resistance Surveillance System (EARSS). J Antimicrob Chemother 48: 143–144. 11418528 10.1093/jac/48.1.143

[38] Biber A, Abuelaish I, Rahav G, Raz M, Cohen L, Valinsky L, et al (2012) A typical hospital—acquired methicillin-resistant *Staphylococcus aureus* clone is widespread in the community in the Gaza strip. PLoS One 7: e42864 doi: 10.1371/journal.pone.0042864 22916171 10.1371/journal.pone.0042864 PMC3420888

[39] Aqel AA, Alzoubi HM, Vickers A, Pichon B, Kearns AM (2015) Molecular epidemiology of nasal isolates of methicillin-resistant *Staphylococcus aureus* from Jordan. J Infect Public Health 8: 90–97. doi: 10.1016/j.jiph.2014.05.007 25002017 10.1016/j.jiph.2014.05.007

[40] Abou Shady HH, Baker A.E.A, Hashad ME, Alzohairy MA (2014) *Staphylococcus aureus* nasal carriage among outpatients attending primary health care centers: a comparative study of two cities in Saudi Arabia and Egypt. Braz J Infect Dis 19: 68–76. doi: 10.1016/j.bjid.2014.09.005 25523075 10.1016/j.bjid.2014.09.005 PMC9425251

[41] Senok A, Somily A, Raji A, Gawlik D, Al-Shahrani F, Baqi S, et al (2016) Diversity of methicillin-resistant *Staphylococcus aureus* CC22-MRSA-IV from Saudi Arabia and the Gulf region. Int J Infect Dis 51: 31–35. doi: 10.1016/j.ijid.2016.08.016 27578204 10.1016/j.ijid.2016.08.016

[42] Monecke S, Coombs G, Shore AC, Coleman DC, Akpaka P, Borg M, et al (2011) A field guide to pandemic, epidemic and sporadic clones of methicillin-resistant *Staphylococcus aureus*. PLoS One 6: e17936 doi: 10.1371/journal.pone.0017936 21494333 10.1371/journal.pone.0017936 PMC3071808

[43] Monecke S, Skakni L, Hasan R, Ruppelt A, Ghazal SS, Hakawi A, et al (2012) Characterization of MRSA strains isolated from patients in a hospital in Riyadh, Kingdom of Saudi Arabia. BMC Microbiol 12: 146 doi: 10.1186/1471-2180-12-146 22823982 10.1186/1471-2180-12-146 PMC3464608

[44] Tokajian ST, Khalil PA, Jabbour D, Rizk M, Farah MJ, Hashwa FA, et al (2010) Molecular characterization of *Staphylococcus aureus* in Lebanon. Epidemiol Infect 138: 707–712. doi: 10.1017/S0950268810000440 20202283 10.1017/S0950268810000440

[45] Coombs GW, Monecke S, Pearson JC, Tan HL, Chew YK, Wilson L, et al (2011) Evolution and diversity of community-associated methicillin-resistant *Staphylococcus aureus* in a geographical region. BMC Microbiol. 11: 215 doi: 10.1186/1471-2180-11-215 21955438 10.1186/1471-2180-11-215 PMC3197503

[46] Chung M, Dickson G, de Lencastre H, Tomasz A (2004) International clones of methicillin-resistant *Staphylococcus aureus* in two hospitals in Miami, Florida. J Clin Microbiol 42: 542–547. doi: 10.1128/JCM.42.2.542-547.2004 14766814 10.1128/JCM.42.2.542-547.2004 PMC344518

[47] Ellington MJ, Yearwood L, Ganner M, East C, Kearns AM (2008) Distribution of the ACME-arcA gene among methicillin-resistant *Staphylococcus aureus* from England and Wales. J Antimicrob Chemother 61:73–77. doi: 10.1093/jac/dkm422 17989100 10.1093/jac/dkm422

[48] Schuenck RP, Nouer SA, Einter Cge O, Cavalcante FS, Scotti TD, Ferreira AL, et al (2009) Polyclonal presence of non-multiresistant methicillin-resistant *Staphylococcus aureus* isolates carrying SCC*mec* IV in healthcare-associated infections in a hospital in Rio de Janeiro, Brazil. Diagn Microbiol Infect Dis 64: 434–441. doi: 10.1016/j.diagmicrobio.2009.04.007 19631097 10.1016/j.diagmicrobio.2009.04.007

[49] Smyth DS, Feil EJ, Meaney WJ, Hartigan PJ, Tollersrud T, Fitzgerald JR., et al (2009) Molecular genetic typing reveals further insights into the diversity of animal-associated *Staphylococcus aureus*. J Med Microbiol 58: 1343–1353. doi: 10.1099/jmm.0.009837-0 19528163 10.1099/jmm.0.009837-0

[50] Sung JM-L, Lloyd DH, Lindsay JA (2008) *Staphylococcus aureus* host specificity: comparative genomics of human versus animal isolates by multi-strain microarray. Microbiol 154: 1949–1959 10.1099/mic.0.2007/015289-0 18599823

[51] Seidl K, Leimer N, Marques MP, Furrer A, Holzmann-Burgel A, Senn G, et al (2015) Clonality and antimicrobial susceptibility of methicillin-resistant *Staphylococcus aureus* at the University hospital Zurich, Switzerland between 2012 and 2014. Annals of Clinical Microbiology and Antimicrobials 14: 14 doi: 10.1186/s12941-015-0075-3 25858549 10.1186/s12941-015-0075-3 PMC4369350

[52] Nubel U, Nachtnebel M, Falkenhorst G, Benzler J, Hecht J, Kube M, et al (2013) MRSA transmission on a neonatal intensive care unit: epidemiological and genome-based phylogenetic analyses. PLoS One 8: e54898 doi: 10.1371/journal.pone.0054898 23382995 10.1371/journal.pone.0054898 PMC3561456

[53] Williams K, Hopkins S, Turbitt D, Seng C, Cookson B, Patel BC, et al (2014) Survey of neonatal unit outbreaks in North London: Identifying causes and risk factors. J Hosp Infect 88: 149–155. doi: 10.1016/j.jhin.2014.06.012 25146223 10.1016/j.jhin.2014.06.012

[54] Takei Y, Yokoyama K, Katano H, Tsukiji M, Ezaki T (2010) Molecular epidemiological analysis of methicillin-resistant staphylococci in a neonatal intensive care unit. Biocontrol Sci. 15: 129–138. 21212505 10.4265/bio.15.129

